# GNOme, an ontology for glycan naming and subsumption

**DOI:** 10.1007/s00216-025-05757-8

**Published:** 2025-02-08

**Authors:** Wenjin Zhang, Michelle Vesser, Nathan Edwards

**Affiliations:** Department of Biochemistry and Molecular & Cellular Biology, Georgetown University Medical Center, Washington, DC USA

**Keywords:** Glycans, Ontology, Subsumption

## Abstract

While GlyTouCan provides stable identifiers for referencing glycan structures, they are not organized semantically. GNOme, a glycan naming and subsumption ontology and a member of the OBOFoundry, organizes GlyTouCan accessions for automated reasoning and interactive browsing of glycan structures by subsumption. GNOme makes it quick and easy to discover glycans with a specific degree of characterization; provides a text-based table of common synonyms for specific structures and compositions; enumerates glycan subsumption relationships for automated reasoning; and assigns each glycan to well-defined categories based on their degree of characterization. As an OBOFoundry ontology, GNOme can be readily integrated with other OBOFoundry ontologies and standards initiatives that need to refer to glycans with various degrees of characterization. GNOme is integrated with GlyGen, a glycoinformatics knowledge base, providing navigation to “related glycans,” and expanding the utility of species and glycan classification annotations. GNOme is available at https://gnome.glyomics.org/ and via GlyGen, the OBO Foundry, and GitHub.

**Graphical abstract:**

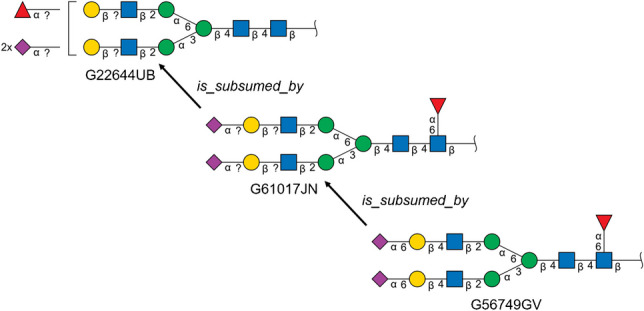

**Supplementary Information:**

The online version contains supplementary material available at 10.1007/s00216-025-05757-8.

## Introduction

An important tool for describing glycan molecules, glycan sequence formats such as GlycoCT [1] and WURCS [2, 3] explicitly specify, or indicate the absence of knowledge about, each detail of a glycan’s structure—this is crucial, since current experimental techniques for characterizing glycans generally do not fully characterize the structures, and these sequence formats capture which details are known or not known. Unfortunately, however, this makes the sequences long and complex, and therefore difficult to communicate or share. GlyTouCan [4] is an international registry that provides stable accessions for glycan sequences, facilitating communication and knowledge sharing. However, as the number of registered glycan sequences in GlyTouCan has grown beyond 200,000, finding appropriate glycan (sequence) accessions is difficult because they are not organized semantically. We propose to organize GlyTouCan accessions by subsumption, that is, with respect to their degree of glycan characterization, with the Glycan Naming and Subsumption Ontology (GNOme). An OBO Foundry [5] ontology, GNOme’s web-based graphical user interface makes it easy to determine glycan structure accessions for glycans with a specific degree of characterization. GNOme provides a text-based lookup for common synonyms for specific structures and compositions. GNOme’s explicit enumeration of glycan subsumption relationships facilitates automated reasoning. Finally, GNOme assigns to glycans to well-defined categories based on their degree of characterization. As an OBO Foundry ontology, GNOme can be readily used by other ontology and standards initiatives, for references to glycans with varying degrees of characterization. GNOme is integrated with GlyGen [6], a glycoinformatics knowledge base, providing navigation to “related glycans,” and expanding the utility of species and glycan classification annotations.

## Materials and methods

### Glycan structure subsumption relationships

The GNOme ontology is computationally determined from the accessions and glycan sequences of GlyTouCan. The structures are grouped first by molecular weight, and then aligned, with respect to the subsumption, within each group.

The subsumption relationship partial order *a* $\supset$ *b*, indicating that glycan *a* subsumes glycan *b*, is determined by enumerating subsumption aware perfect matchings between the monosaccharides of *a* and *b*. If the subsuming glycan *a* is a composition, with no glycosidic links, then any such matching is sufficient to establish the subsumption relationship. If the subsumed glycan *b* is a composition and *a* is not, then *b* is not subsumed by *a*. For each subsumption aware perfect matching between the monosaccharides of the glycan structures *a* and *b*, then, we must next check for a subsumption aware matching of the links between matched monosaccharides. All links from the subsumed structure *b* must be subsumed by a link the subsuming structure *a*, but some undetermined links from the subsuming structure *a* can remain unmatched (see Supplementary Material). Floating substituents in the subsuming structure may also need to be matched to monosaccharide-attached substituents in the subsumed structure. Tree-based backtracking combinatorial enumeration algorithms are used to find valid monosaccharide perfect matchings and link matchings for subsumption. This graph isomorphism-style combinatorial strategy is necessary to support glycan compositions and structures with undetermined linkages—such structures can no longer be treated as trees (a common representation for topologically determined glycans) and must be aligned as directed graphs.

To accommodate alditol-reduced reducing-end glycan structures, we (redundantly) add such structures to the unreduced reducing-end structures’ molecular weight group before alignment—enabling subsumption relationships between unreduced reducing-end structures and alditol-reduced reducing-end structures.

Once pairwise subsumption relationships have been computed for all structures in a molecular weight group, we remove any relationship that can be inferred by transitivity. The resulting minimal set of subsumption relationships form the basis of GNOme. The computational approach for parsing GlyTouCan glycans, grouping by molecular weight, manipulating structures, and subsumption alignment is implemented in the PyGly Python package available from the GitHub repository https://github.com/glygen-glycan-data/PyGly.

### Characterization levels and archetypes

GNOme defines five subsumption-based characterization-level categories. In most well-characterized to least well-characterized order, these are Saccharide, Topology, Composition, BaseComposition, and Molecular Weight. These characterization levels are based on the ROCS RDF ontology [7] developed by the GlyTouCan project. The presence or absence of specific structural information in the glycan sequence drives the subsumption category assignment for each structure. The molecular weight category is not part of ROCS, but the molecular weight can be readily computed from the structure description, and it conceptually subsumes base compositions. In addition, many experimental strategies for glycan characterization only identify a structure's molecular weight, and from that, infer base composition. Removal of specific types of structural information from a glycan sequence results in a less well-characterized glycan structure, potentially belonging to subsumption category for less well-characterized structures. Table 1 shows the categories and the specific information whose absence defines membership in each category.

**Table 1:** GNOme subsumption categories and missing structure information. Y indicates the presence of one of more elements with the indicated structure information; “-” indicates the complete absence of the indicated structure information

Category	Superclass	Stereochemistry	Glycosidic links and carbon ring	Anomeric config. and carbon bonds
Molecular weight	-	-	-	-
BaseComposition	Y	-	-	-
Composition	Y	Y	-	-
Topology	Y	Y	Y	-
Saccharide	Y	Y	Y	Y

Each of the levels Topology, Composition, and BaseComposition can also be considered as an operation, in which the structural information not relevant for the level is removed. If, after application of these operations, the structure is unchanged, then structure itself can be assigned to that level. When a structure is changed by the application of these operations, the new structure is *the* topology, composition, or base composition form of the structure. These characterization level structure relationships allow structures to be grouped by common topology or composition. We note that the characterization level structure relationships may skip many intermediate subsumption relationships, and as such they can be considered “shortcuts” to topologies, compositions, and base composition structures within the ontology.

Recently, the GlyTouCan project proposed the notion of Archetype structures, in which the reducing-end monosaccharide only has anomeric and ring-information removed, collapsing redundancy due to varying details in anomeric and ring-information characterization at glycans’ reducing-ends. As with the characterization levels, we treat Archetype as an operation which either establishes the structure is an Archetype or indicates the related Archetype structure, if present in GlyTouCan, for any given structure.

### Composition strings and semantic names

Glycan structures with their WURCS strings are checked to determine whether they represent compositions and whether their WURCS monosaccharide residue codes represent supported composition string monosaccharides: Hex, HexNAc, Fuc, dHex, NeuAc, NeuGc, KDN, HexA, Pent. If so, composition strings are generated in Byonic [8], single letter, and so-called UniCarbKB [9] formats. Each such composition string is then associated with its GlyTouCan accession and annotated in GNOme.

### Structure characterization scores

A heuristic structure (lack of) characterization score between 0 and 10,000 is computed for each GNOme structure. Fully determined structures have characterization scores of 0. Base compositions have characterization scores of 10,000. To compute the characterization score of a glycan structure, each monosaccharide is scored first, and the *n* monosaccharide scores are averaged. The *n*−1 conceptual links between non-reducing-end monosaccharides and their parent monosaccharides are considered next, making it possible to score the lack of information about the attachment of the monosaccharides, even if there is no link (composition, base composition) or undetermined linkage topology. The conceptual links’ scores are then averaged. Finally, the average monosaccharide score and average link score are subject to a weighted sum, with the monosaccharide score representing ~ 40% and the link score representing ~ 60% of the final characterization score (see Supplementary Material). This scheme produces quite distinct and interpretable scores regimes for structures in the BaseComposition, Composition, Topology, and Saccharide subsumption categories. The score is monotonic with respect to the subsumption relationships between structures, so that a subsumed structure always has a smaller score. Scores of structures with different compositions or those which do not have a subsumption relationship are not guaranteed any specific ordering, but those with qualitatively different degrees of characterization usually receive scores that correctly order the structures. As such, the (lack of) characterization score can be used to sort unrelated glycans to achieve a crude ordering from most to least characterized.

### Formal ontology structure

The ontology uses GlyTouCan accessions to define its primary terms, as a RDF Schema *class*, with the required OBO Foundry term naming structure (e.g., GNO_G00912UN). Terms published in GNOme that are based on accessions subsequently archived or replaced by GlyTouCan are marked as obsolete and indicate the replacement accession where possible. Terms representing other classes, such as the glycan root and molecular weight groups are assigned permanent number-based terms.

The GNOme ontology is a member of the OBO Foundry with prefix GNO and released as an OWL-format ontology with automatically generated OBO and JSON derived formats using the robot tool [10]. In addition to GNOme classes for each supported GlyTouCan accession, GNOme creates molecular weight class terms, to represent the molecular weight grouping of subsumption relationships (to two decimal places), and a root Glycan class that subsumes each of the molecular weight terms. The *subClassOf* predicate (from the RDF Schema) is used to represent the primary (mass-preserving) subsumption relationships. An additional predicate *is_subsumed_by*, is used to represent both mass-preserving and non-mass-preserving subsumption relationships (which includes alditol-reducing reducing-end structure subsumption relationships with non-reduced reducing-end structures). The subsumption relationships define a directed acyclic graph, rather than a tree, since a given structure may have multiple subsuming structures, even after relationships that can be inferred by transitivity have been removed.

GNOme defines a variety of specific annotation properties and predicates for each class term representing a GlyTouCan accession and its sequence:

- *subClassOf*: GNOme URI of subsuming structure(s), inclusive of mass-preserving subsumption relationships only.
- *is_subsumed_by*: GNOme URI of subsuming structure(s), inclusive of mass-preserving and non-mass-preserving subsumption relationships.
- *has_glytoucan_id*, *has_glytoucan_link*: GlyTouCan accession and deep linking URL to the corresponding GlyTouCan webpage.
- *has_subsumption_category*: GNOme URI of the subsumption category.
- *has_basecomposition*; *has_composition*; *has_topology*: GNOme URI of the structure with the appropriate information removed, if it exists.
- *has_structure_browser_link*, *has_composition_browser_link*: URL of deep link to interactive GNOme browser web-applications for structures and compositions, where appropriate.
- *has_Byonic_name*, *hasExactSynonym*: Synonyms for the GNOme term, including specific predicate for composition strings as formatted by the Byonic glycopeptide identification search engine. *hasExactSynonym* is defined in the oboInOwl ontology.
- *has_structure_characterization_score*: The structure characterization score.
- *is_restriction_member*: Boolean indicating whether the accession is a member of the restriction set (only present in restrictions—see “Glycan structure restriction sets” below).

Other properties used by GNOme include predicates from the oboInOwl ontology (*hasExactSynonym*), the *definition* property from the Information Artifact Ontology, and other Web Ontology Language terms, including *label*. GNOme strives to meet and abide by the OBO Foundry principles, being consistent with other OBO Foundry ontologies to promote reuse, and is integrated with ontology support services, such as OntoBee [11] and OLS [12].

### Glycan structure restriction sets

In order to support glycoinformatics resources that capture a subset of GlyTouCan’s structures, GNOme also releases ontology restrictions for subsets of GlyTouCan accessions, in which only the relevant GNOme terms are retained and direct subsumption relationships between restriction members are inferred by transitivity from the primary ontology. GNOme supports ontology restrictions for GlyGen, GlyCosmos [13], BSCDB [14], and PubChem Compound [15].

In addition to these glycoinformatics resources, we support *semantically* constrained restrictions for N-linked and O-linked glycan structures as defined by various GlyGen affiliated projects. The N-linked glycan restriction constrains glycans to those with the N-glycan core motif, defined to be those GlyTouCan structures with core-alignment to GlycoMotif motif N-Glycan core basic (GGM.001001). The GlyGen N-linked and O-linked glycan restriction constrains glycans to those structures in GlyGen that are classified as N-linked or O-linked (also based on GlycoMotif core-alignments). Finally, the GlyGen GlycoTree Sandbox N-linked and O-linked glycan restrictions constrain glycans to those GlyGen structures that completely align to the manually curated N-linked and O-linked mammalian glycan super-trees of the GlycoTree Sandbox. Since the GlycoTree Sandbox can only completely align fully defined structures to the glycan super-trees, the GlycoTree Sandbox restriction also includes all GNOme ancestors of GlycoTree Sandbox glycans.

In addition to the glycan structures considered to be members of the restriction, we also add archetypes, topologies, compositions, and base compositions for each structure to the restricted ontology to aid in navigation within the ontology and the graphical user interface. Structures that are members of the restriction are annotated with the *is_restriction_member* property.

### Graphical user interface

The GNOme ontology, unlike other human-curated ontologies of conceptual terms defined by English language definitions, has terms that are essentially uninterpretable. In order to enable ontology users to browse and explore glycans by subsumption, existing text-based tools were not sufficient, instead a graphical user interface based on glycan images or cartoons was needed. We implemented a JavaScript-based widget and website (https://gnome.glyomics.org) that reads a GNOme-derived JSON document and presents a visual, interactive, and navigable portal into glycan subsumption. The graphical user interface provides a natural way for glycobiologists, glycoinformaticists, and others to navigate to specific glycan structures, with the correct level of characterization, that match the experimental data at hand. The GNOme JavaScript widgets use the vis.js and jquery.js JavaScript libraries for the graph-based layout and asynchronous data-retrieval tasks, respectively. The GNOme website and web-applications are served out of the GNOme GitHub repository: https://github.com/glygen-glycan-data/GNOme.

### SNFG consistent glycans

In order to characterize the extent to which GNOme covers useful glycan sequences registered in GlyTouCan, we used the monosaccharides, in their most common configuration, and substituents of the Symbol Nomenclature for Glycans (SNFG) [16] Discussion Group as a surrogate for the most commonly used monosaccharides and substituents. SNFG Consistent Glycans are those that consist entirely of monosaccharides and substituents consistent with this SNFG set (see Supplementary Material). A SNFG monosaccharide table, with associated WURCS sequences, is provided at the GNOme website https://gnome.glyomics.org/SNFG, while the SNFG consistency of each monosaccharide and unattached substituent in each GlyTouCan WURCS sequence is provided with each GNOme release at https://gnome.glyomics.org/data/glytoucan_snfg.txt.

## Results

The GNOme ontology (release 2.2.0, May 21, 2024) defines 15 annotation properties, 5 named instances (subsumption categories), and 191,943 classes. The classes are made up of 1 root glycan class, 1 subsumption category class, 14,977 molecular weight classes, and 176,965 classes representing GlyTouCan accessions, of which 7705 are marked obsolete and represent archived, replaced, or missing GlyTouCan accessions and 169,212 represent current GlyTouCan accessions.

Total wall-clock time for single CPU computation of subsumption alignments for release 2.2.0 was about 18 h on a Dell PowerEdge 2950-III dual quad-core Xeon X5460 @ 3.17MHz running CentOS Linux 7.9.

### Coverage of GlyTouCan glycans

As of May 2024, GlyTouCan reports 247,704 glycans on its website, of which 246,858 are not marked archived or replaced by GlyCosmos. GlyCosmos has 243,302 accessions not marked as archived or replaced. GNOme’s GlyTouCan accession terms represent 68.5% of the current GlyTouCan glycan set, and 69.5% of the GlyCosmos set. Some of the missing accessions are glycans with repeating substructures (9876), which GNOme does not currently support. For the rest, we have established that many of these structures describe esoteric monosaccharide entities, chemically modified glycans, or substituent-modified linkages.

In order to quantify the extent to which GNOme captures important glycan structures in common use, we tested each WURCS sequence against the Symbolic Notation for Glycans (SNFG) project monosaccharides and substituents, marking those that consist entirely of SNFG consistent components. Of the 117,737 GlyTouCan accessions without repeating substructures that meet this criterion, 99.99% are represented in GNOme, with just six accessions not supported. A manual analysis confirms the WURCS sequences of these six accessions are likely incorrect. We note that GNOme supports an additional 51,481 accessions beyond this SNFG consistent glycan set.

For a related test of GNOme coverage, we used the GlycanBuilder2 tool [17], developed in the same lab as the WURCS Framework and using software infrastructure developed by the same group, to establish a set of good GlyTouCan glycans. GlycanBuilder2 can parse approximately 75.5% of GlyTouCan sequences, and of these, 88.8% are supported by GNOme.

### Subsumption categories and archetypes

The GNOme ontology (release 2.2.0, May 21, 2024) assigns 87,182 glycans to the Saccharide subsumption category, 53,200 glycans to the Topology category, 19,779 glycans to the Composition category, and 9051 glycans to the BaseComposition category. Of these, 27,682 of the Saccharide glycans has at least one of its corresponding Topology, Composition, or BaseComposition glycans missing from the supported GlyTouCan set, while 59,500 Saccharide glycans have a complete set of corresponding Topology, Composition, or BaseComposition glycans in GNOme. Table 2 provides some statistics on the subsumption categories.

**Table 2:** Counts and characterization scores of glycans in each subsumption category. Minimum and maximum possible scores are 0 and 10,000

		Characterization scores		
Category	Count	10^th^ percentile	Median	90^th^ percentile
BaseComposition	9051	7979	10,000	10,000
Composition	19,779	1969	8030	9343
Topology	53,200	1641	1969	2385
Saccharide	87,182	0	164	984
Archetype	44,722	-	-	-

A total of 44,722 glycans match the criteria for annotation as archetypes, where the reducing-end monosaccharide has no anomeric or ring information, while 67,938 glycans subsumed by these archetypes are annotated to indicate their archetypes. Each archetype represents an average of about 2.3 structures, including itself. Several single-monosaccharide archetype glycans represent the most structures (14), but considering multi-residue archetype glycans only, G61305ZB represents the most structures (9). There are 4763 archetypes that represent only themselves. There are 4125 alditol-reduced reducing-end Saccharide or Topology glycans with an archetype representative and 3920 without. A total of 31,592 Saccharide or Topology glycans do not have an annotated archetype and are not an archetype themselves.

Ongoing efforts to register the Topology, Composition, BaseComposition, and Archetype forms of existing GlyTouCan structures are expected to fill these gaps over time.

### Characterization score

**Fig. 1:**
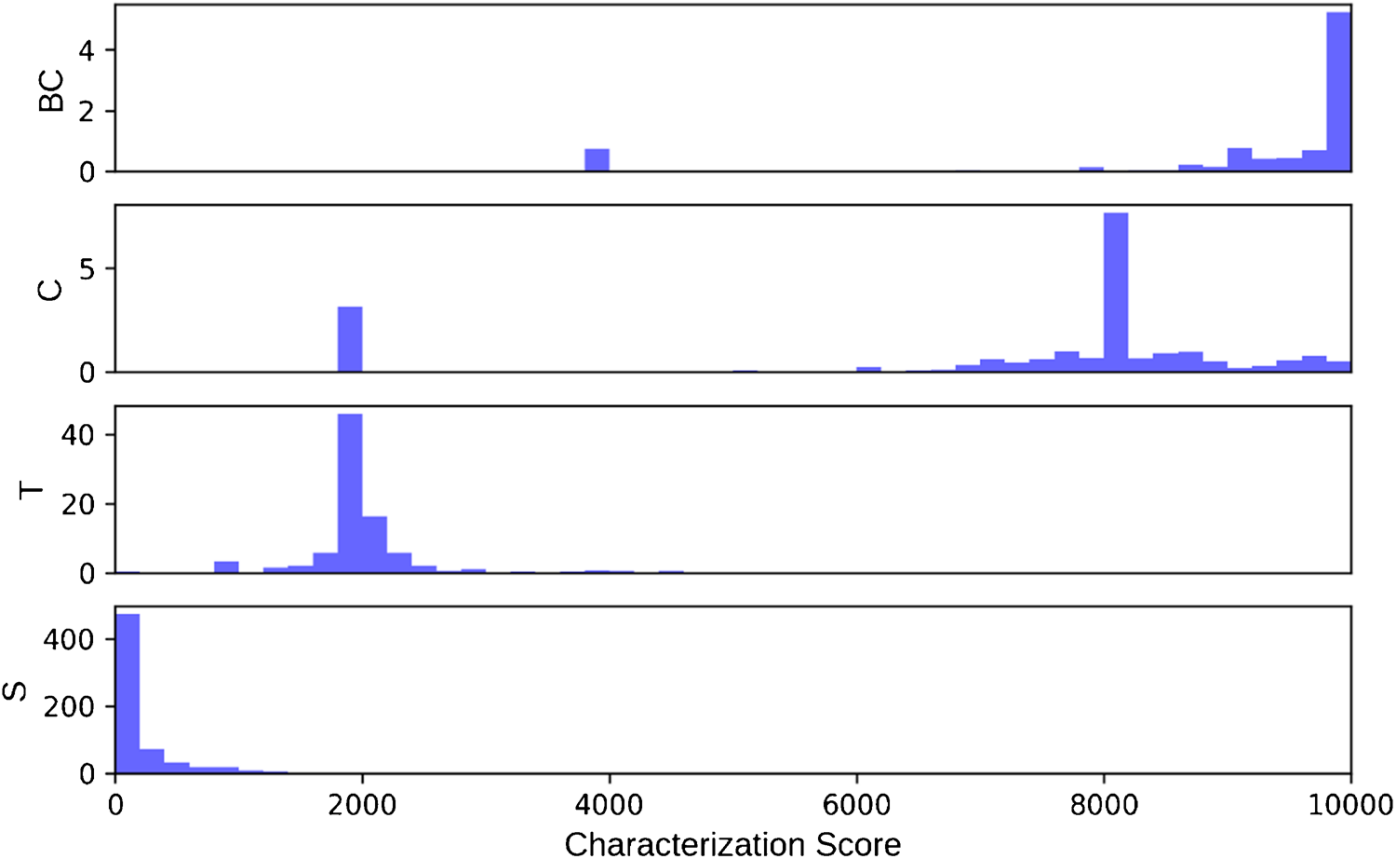
Distribution of characterization scores for Saccharide (S), Topology (T), Composition (C), and BaseComposition (BC) subsumption categories

The (lack of) characterization score is designed to allow structures to be sorted numerically from most completely characterized to least completely characterized. We determined the need for a scoring regime which (a) respected subsumption; (b) correctly ordered glycans with significantly different degrees of characterization, including undetermined topologies, compositions, and fully defined glycans, whether they have a similar number of monosaccharides; and (c) gave similar scores to structures with the same topology and a similar degree of characterization. The weighting of the different components of the score and the evaluation of whether these objectives have been met is quite subjective; nevertheless, we feel the current GNOme characterization score has good utility. Table 2 and Fig. 1 show the distribution of characterization scores for glycans in each of the subsumption categories. Glycans from the Saccharide and BaseComposition categories have 0, representing a fully defined glycan structure, and 10,000, representing a monosaccharide (base-)composition with no stereochemistry, ring, linkage, or anomeric configuration, as their most common and median scores respectively. Topology category glycans have median scores around 2000, while Composition glycans have median scores around 8000.

**Fig. 2:**
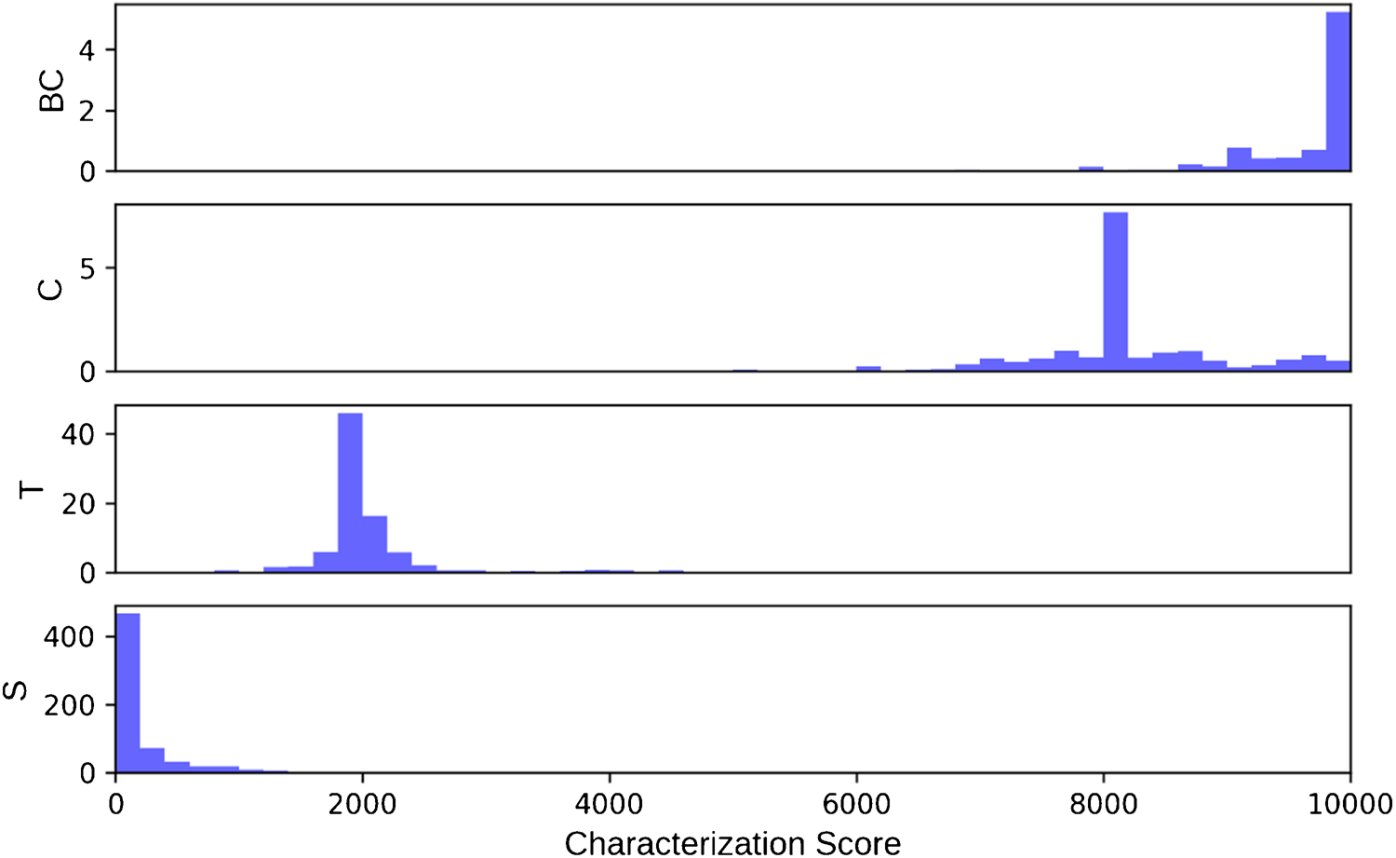
Distribution of characterization scores for Saccharide (S), Topology (T), Composition (C), and BaseComposition (BC) subsumption categories with single-monosaccharide glycans removed

While, in general, the scores of glycans in different categories are well separated, there are exceptions. Many monosaccharide compositions mix monosaccharides with, such as Neu5Ac, and without, such as HexNAc, stereochemistry information. A Saccharide glycan structure might have little linkage and anomeric information and many residues with undetermined linkage, moving its score closer to Topology glycans. Finally, single monosaccharides are difficult to categorize. They have no glycosidic linkages, but the presence of carbon-ring information or anomeric configuration, or the absence of stereochemistry information indicates weak membership in one of the subsumption categories. Nevertheless, these single-monosaccharide glycans create an observable blip in the BaseComposition score distribution, around 4000, and the Composition score distribution, around 2000 (see Fig. 1). This makes some sense, since the links which distinguish a Topology glycan from a Composition glycan are not present. Figure 2 shows the distribution of characterization scores with single-monosaccharide glycans removed.

### Graphical user interface

**Fig. 3:**
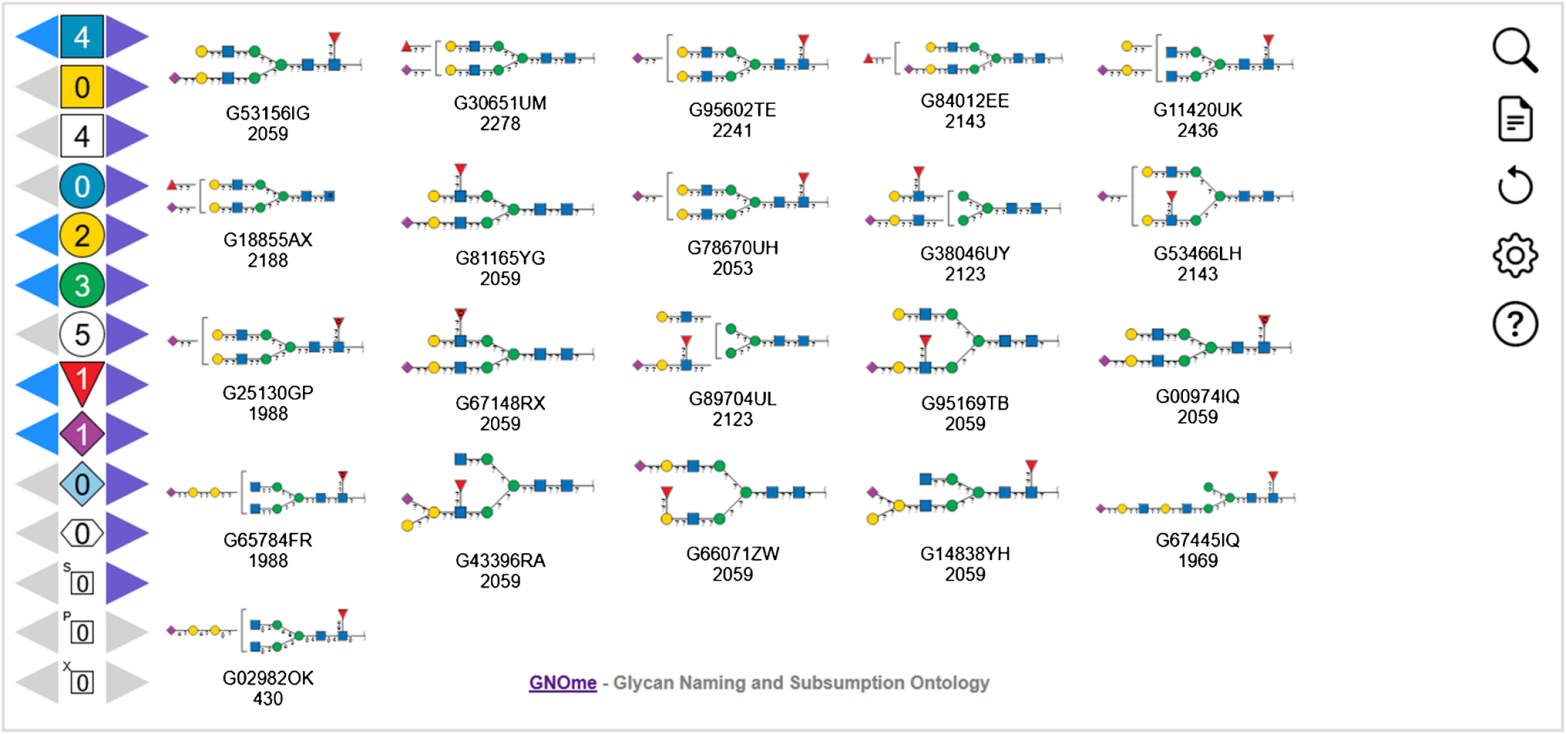
GNOme Browser’s Topology Selector showing topologies consistent with monosaccharide composition GlcNAc(4)Gal(2)Man(3)Fuc(1)NeuAc(1)

**Fig. 4:**
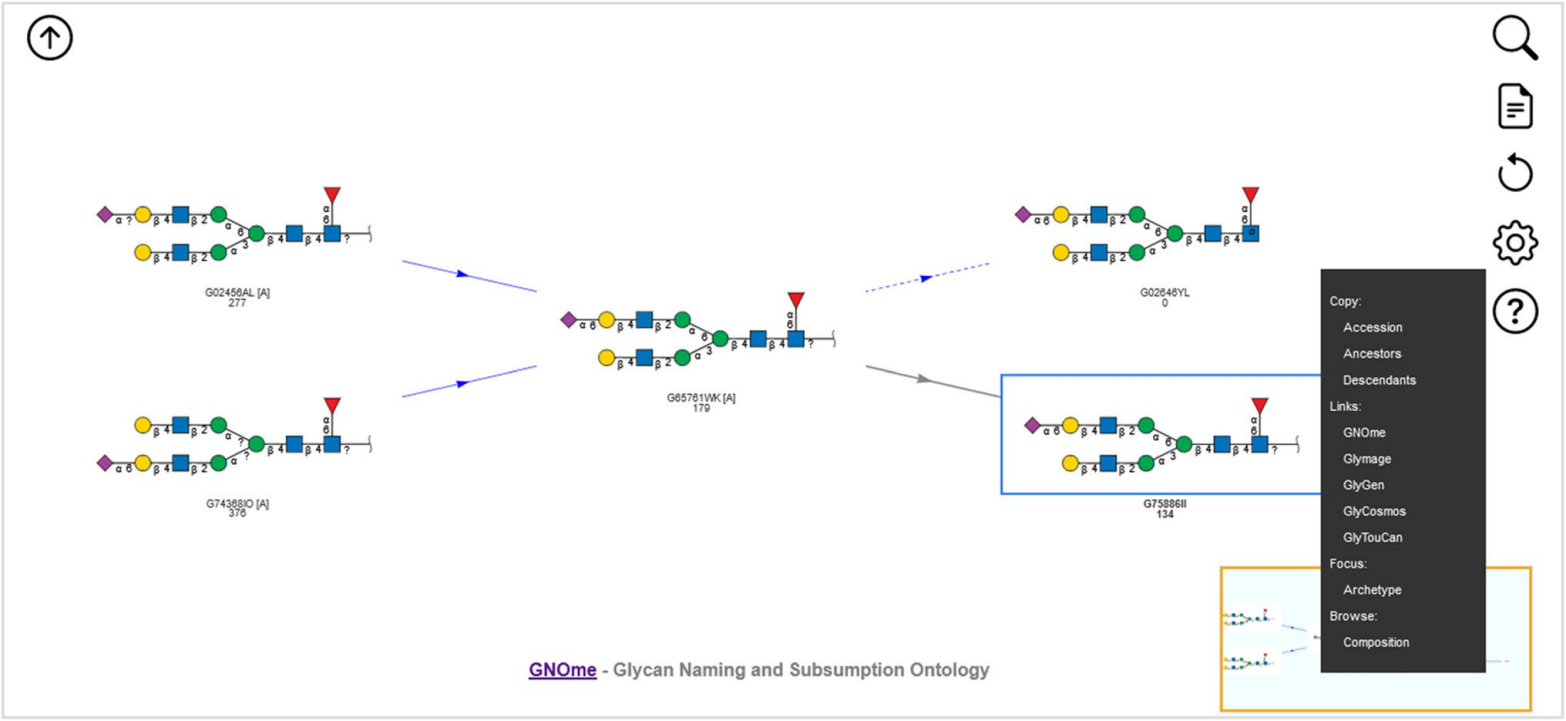
The GNOme Browser’s Subsumption Navigator focused on GlyTouCan accession G65761WK, subsumed by G02456AL and G74368IO. Structure G65761WK subsumes G02646YL and G75886II. Structure G02646YL, shown with a dashed edge, represents an alditol-reduced reducing-end structure with a different molecular weight than G65761WK. Optional characterization scores and archetype markers also shown. Popup menu provides options for copying the accession, ancestor or descendant accessions to the clipboard; links to various related resources, including the OntoBee presentation of GNOme terms, Glymage SVG image, GlyGen, GlyCosmos, and GlyTouCan; refocusing on the Archetype glycan; and jumping to the composition in the GNOme Composition Browser

The GNOme browser consists of two navigation panels, the Topology Selector, see Fig. 3, and the Subsumption Navigator, see Fig. 4. The Topology Selector panel presents the user with buttons representing common monosaccharides and substituents, so that a monosaccharide composition of interest can be selected. Relevant topologies are updated in real-time, ordered left-to-right by the number of subsumed descendants for each topology. GlyTouCan accession and, optionally, characterization score, an archetype marker, and restriction membership are shown under each topology. Only those topologies not subsumed by some other topology are shown. Clicking on a topology structure shifts focus to that topology in the Subsumption Navigator. Figure 3 shows topologies consistent with the monosaccharide composition GlcNAc(4)Gal(2)Man(3)Fuc(1)NeuAc(2). The HexNAc button adds any HexNAc, similarly for the Hex button. As such, selecting HexNAc(4)Hex(5)Fuc(1)NeuAc(2) would also show these topologies and more. Importantly, a mixture of stereochemistry-specific and stereochemistry-non-specific HexNAc and Hex monosaccharides can be specified, if desired. Sulfate and phosphate substituents can also be specified, whether attached to a monosaccharide or not. Finally catch-all monosaccharide and substituent buttons are provided to add residues and substituents that cannot otherwise be chosen. The browser URL is updated to reflect the button state as they are changed and can be bookmarked or sent to others. See also deep-linking below.

The Subsumption Navigator focuses on a specific structure, chosen in the Topology Selector, and is shown in the center of the panel. Subsuming structures are shown on the left, with arrows towards the focus structure, arrayed top-to-bottom in increasing characterization score order (most characterized to least). Subsumed structures are shown on the right, with arrows pointing away from the focus structure, arrayed top-to-bottom in increasing characterization score order. Double-clicking on any subsumed or subsuming structure will re-focus the Subsumption Navigator. To the right of each subsumed structure is an indication of how many structures it subsumes, if any. Dashed arrows indicate subsumption relationships that do not preserve molecular weight, which currently are used to represent alditol-reduced reducing-end structures’ relationships to non-reduced reducing-end structures. The GlyTouCan accession and, optionally, characterization score, an archetype marker, and restriction membership are shown under each structure. The browser URL is updated to reflect the focus structure as it changes and can be bookmarked or sent to others (see also deep-linking below). A return button (up arrow icon) is shown at the top-left of the Subsumption Navigator to return to the Topology Selector panel, with corresponding topologies highlighted.

A popup menu is available by right-clicking on each structure in the Subsumption Navigator. The popup menu provides various options depending on the structure’s accession. First, there are options for copying the structure’s accession, ancestor or descendant accessions, to the clipboard. Second are external link options including the OntoBee term page, the Glymage SVG image, GlyTouCan, GlyCosmos, and GlyGen, where applicable. In the restrictions, additional external link options are displayed, to PubChem or the GlycoTree Sandbox site. Third, when the structure has an archetype structure associated with it, the option to refocus the Subsumption Navigator on the archetype structure is provided. Finally, a link to jump to the Composition Browser’s Composition Selector page with the corresponding monosaccharide button state selected.

Several navigation buttons are always shown on the top-right of the display: Find (magnifying glass icon), Align (notepad icon), Reset (recycle icon), Settings (cog icon), and Help (question mark icon). Find will look-up a GlyTouCan accession or semantic name and focus the Subsumption Navigator on the corresponding accession, if it is a topology or saccharide, or preselect the corresponding monosaccharide button state, if the accession is a composition or base composition. Align calls the subsumption webservice at https://subsumption.glyomics.org/ to place a (potentially) novel WURCS or GlycoCT sequence in its subsumption relationship with GNOme terms representing GlyTouCan glycans. If the sequence represents an existing GNOme term, the behavior is similar to the Find option, but if the sequence is novel, it is shown as a new node, with glycan image computed on the fly using the Glymage webservice https://glymage.glyomics.org/, and labeled “Query,” as the current focus accession. Reset sets the monosaccharide button state back to initial values and removes any Align structure from the browser. Settings configures the optional displayed values under each structure in the two panels—whether to display the characterization score (default: False), whether to display the archetype marker “[A]” (default: True), and whether to display the restriction membership marker “*” when using a restriction browser (default: True). Finally, the Help button brings up brief panel-specific help and provides a link to the documentation provided in the GitHub repository.

The GNOme Structure Browser supports deep linking for integration with other glycoinformatics resources and tools. Using a “focus” URL parameter with a GlyTouCan accession with behave as if the accession was entered using the Find button. When the accession represents a composition or base composition, the user will be taken to the Topology Selector panel, with corresponding monosaccharide buttons set. When the accession represents a topology or saccharide structure, the user will be taken to the Subsumption Navigator, focused on the accession. Similarly, the Structure Browser supports the addition of IUPAC symbols, representing the various monosaccharide buttons, as URL parameters, and resulting in the Topology Selector panel, with monosaccharide buttons set appropriately. The URL parameters for deep linking match those in the updated URL as the Topology Selector monosaccharide buttons are manipulated, or the Subsumption Navigator focus is changed, so an appropriate URL for deep linking can be readily determined.

### Applications and integrations

The GNOme ontology has found application and been integrated with the GlyGen glycoinformatics data-resource, with other ontologies and OBO Foundry resources, and with open-standards file-formats.

#### Integration with GlyGen

The GNOme ontology was developed under the banner of the GlyGen: Computational and Informatics Resources for Glycoscience project to solve fundamental glycoinformatics sticking points for glycan structure repositories. First, curated glycan annotations such as a glycan’s species, extracted from published manuscripts, might be attached to GlyTouCan accessions at various places in the subsumption hierarchy, despite representing otherwise consistent experimental observations. Species annotations derived from mass spectrometry analyses are typically associated with monosaccharide compositions (or base compositions), while more detailed, painstaking biochemical analyses coupled with prior glycobiology knowledge might lead to the determination of specific topological or glycosidic linkage characteristics. While the glycobiology community is quite comfortable with switching between compositions and structures and using semantic context to understand what is implied, glycoinformatics resources must attach annotations to a specific accession. GNOme is used to propagate annotations, particularly species annotations, from more highly characterized structures to the glycans that subsume it. The annotation on a glycan accession represents the concept that at least one concrete glycan structure subsumed by the accession exists in a specific species. GNOme-based species annotations in GlyGen are indicated with an appropriate badge (“Subsumption”) and a link to the subsumed accessions that provide the species annotation.

Similarly, GlyGen propagates glycan type and subtype annotations associated with a glycan structure to those accessions that subsume it. This makes it possible to find monosaccharide compositions that subsume a glycan subtype assigned due to a specific structural characteristic—such as core-fucosylation. Together, these annotations enable the construction of a Byonic-format file of 369 human N-linked glycan monosaccharide compositions with embedded GlyTouCan accessions for deep-linking to GlyGen or GNOme.

Furthermore, GNOme-based annotations of a structure’s topology, composition, and base composition glycans aid with navigation to less well-characterized versions of a structure, which may, by subsumption, have more annotations; while a list of descendants shows the most well-characterized glycans subsumed by a structure. These links in the Subsumption section of the glycan details page help quick navigation up and down the subsumption hierarchy.

More detailed subsumption navigation and browsing is available from the “Related Glycans” button at the top of the page that deep-links into the GlyGen restricted GNOme graphical user interface. Once a specific glycan structure is found in GlyGen restricted GNOme GUI, the popup menu provides a deep-link back to the appropriate GlyGen glycan details page. The (empty) GNOme landing page for the GlyGen restricted GNOme graphical user interface is also available from the GlyGen homepage as the “Structure Browser” card.

#### OBO Foundry integration and GNOme references

The GNOme ontology has been implemented as part of the OBO Foundry ontology infrastructure, providing an ontology landing page; permanent, consistent URLs for the latest version(s) of the ontology in various formats; and links with tools such as OntoBee and OLS for browsing and navigating GNOme as a text-based ontology. As part of the OBO Foundry, GNOme is available for other ontologies and standards to reference alongside other member ontologies, such as the Gene Ontology (GO) [18], the Disease Ontology (DO) [19], and the Human Phenotype Ontology (HPO) [20].

The OBO Foundry protein ontology PRO [21] references GNOme terms extensively, in order to accurately describe the degree of characterization of experimentally described glycans attached to proteins. PRO currently contains 10,010 terms representing glycans attached to proteins. For example, the PRO ID PR:000050940 represents the N-glycosylation of human ceruloplasmin (UniProt accession: P00450) at Asn-138 with a glycan described by GlyTouCan accession G00912UN (GNO:G00912UN), also known as the monosaccharide composition HexNAc(4)Hex(5)NeuAc(2).

GNOme has also been referenced by the Chemical Entities of Biological Interest (ChEBI) [22] OBO Foundry ontology, which uses GNOme to define topology (CHEBI:167,503), composition (CHEBI:167,502), and base composition (CHEBI:167,481) terms under its partially defined glycan term (CHEBI:146,306).

#### Integration with GlyCosmos

The GNOme structure browser is available from GlyCosmos, as a Glycan Search option. Unlike the current loose integration (by separate browser tabs) with GlyGen, GlyCosmos has implemented GNOme by embedding it in a GlyCosmos page. The GNOme JavaScript codebase has been designed to support this use-case, though the URL-based functionality is lost.

#### Proteomics Standards Initiative mzIdentML integration

A current proposal for expanding the Proteomics Standards Initiative (PSI) [23] mzIdentML [24] definition to support glycopeptide identification uses GNOme as one method for referring to glycans attached to peptide substrates. This is particularly natural, as the mzIdentML standard already uses the PSI-MOD [25] OBO Foundry ontology to describe other post-translational modifications observed by tandem mass-spectrometry. The GNOme composition strings provide a simple way for glycopeptide software developers to lookup the GlyTouCan accession and GNOme term associated with a monosaccharide composition.

## Discussion

In its current form, the GNOme ontology has met its original goal of organizing the accessions of GlyTouCan by subsumption for automatic reasoning, such as the propagation of species annotations and glycan types and subtypes to less well-characterized structures and compositions, and for interactive browsing in the GNOme Structure Browser, to find structures with a specific degree of characterization. Its deep-linking capabilities have enabled the straightforward, loose integration of the Structure Browser with GlyGen, while the GlyCosmos site has chosen to embed the Structure Browser directly.

Several unexpected use-cases have been discovered as GNOme has matured. The Byonic glycan-composition set for human N-linked glycans can be readily generated from GlyGen human N-linked annotated glycan structures (and compositions) by traversing GNOme subsumption relationships to less well-characterized glycans until an appropriate GNOme term, with predicate *has_Byonic_name*, is found and added to a non-redundant collection of composition strings, with associated GlyTouCan accessions. This Byonic human N-linked glycan database is available from the GlyGen-Glycan-Data GitHub repository (https://github.com/glygen-glycan-data/PyGly) in the folder smw/glycandata/export with filename byonic_glygen_human_nlinked.txt. This file is regenerated with each GlyGen-Glycan-Data release.

Another unexpected use-case has come from attempts to extract semantic glycan structures from glycan images. In these images, the extraction of carbon bond positions and anomeric configurations was beyond scope for our initial experiments, instead, we focused on extracting topological information: monosaccharides, whether two monosaccharides have a glycosidic linkage, and which monosaccharide is the reducing-end of the structure. Done correctly, this is enough to establish the topological form of the glycan. To assist the curators of manuscripts containing such glycan images, we deep-link into the GNOme Structure Browser based on the extracted topology glycan. First, we check whether the topology glycan has been previously registered with GlyTouCan using the GlyLookup webservice, if so, direct deep-linking via the GlyTouCan accession to the corresponding glycan in the Subsumption Navigator can be carried out. If the topology glycan is not in GlyTouCan, we use the On-Demand Subsumption webservice to place the unregistered topology glycan in the GNOme subsumption hierarchy, thereby identifying the most closely related registered structures, and, again, providing the curator with a quick way to navigate to the specific glycan structure of interest. Finally, if only the monosaccharide composition can be reliably extracted from the image, the GNOme Topology Selector monosaccharide buttons can be set appropriately for the curator to select the topology, and then the detailed structure of interest.

There are several straightforward improvements and extensions to the current GNOme infrastructure that we plan to incorporate in the short-to-medium term. An additional subsumption level predicate *has_ms_composition* is planned to assist with automated reasoning for compositions in which the hexose, *N*-acetylated hexose, deoxyhexose, hexosamine, and hexuronate residues are replaced by Hex, HexNAc, dHex, HexN, and HexA. This structure characterization level is somewhere in-between the current Composition and BaseComposition subsumption levels, since *N*-acetylneuraminic acid, *N*-glycolylneuraminic acid and the other sialic acids have different molecular weights and are not reduced to their stereochemistry-free form.

We plan to more tightly integrate GNOme’s Structure Browser user interface with the GlyGen glycoinformatics portal. The current loose integration was an excellent way to decouple development for both projects initially, but now that the GNOme user interface has matured and stabilized, a more seamless application of subsumption navigation capabilities seems readily within reach, even though some of the benefits of deep-linking would be lost.

Further expansion of the GlyTouCan structures analyzed and organized by GNOme is an ongoing and continual process. We anticipate this will continue for the foreseeable future. While the current glycan data-structures underpinning GNOme are based on GlycoCT concepts, the adoption of a hybrid WURCS/GlycoCT internal monosaccharide representation might permit better coverage of structures containing some of the less commonly used monosaccharides.

More substantial improvements and extensions would be necessary to support an expansion of the subsumption concept to structures described using repeating substructure notation and between structures with additional substituents and even monosaccharides. Conceptually, glycans described using repeating substructures lack the characterization of the number of monosaccharides in the structure, and the specification of the number of repeats will result in a structure that may be semantically equivalent to an existing non-repeating substructure glycan. This conceptual extension to subsumption is expected to be quite difficult to support but may be added to the development roadmap if there is sufficient community interest.

The adoption of subsumption relationships between aditol-reduced reducing-end structures and those with non-reduced reducing-ends is significant because these structures have different molecular weights. It is conceptually reasonable since the aditol-reduced reducing-end is, in some sense, more characterized than a structure with unknown anomeric configuration at the reducing-end and differently characterized than a structure with an alpha or beta anomeric configuration. It would be possible to support relationships representing the addition of one or more phosphate or sulfate substituents, or one or more monosaccharides, but it is unclear whether this fits the subsumption concept—does the addition of a phosphate make the structure more well-characterized? Nevertheless, such relationships would make the user interface more useful and ease the task of finding related glycans. Perhaps a new type of relationship could be added to the ontology to support the user interface in this dimension.

## Conclusion

GNOme, the Glycan Subsumption and Naming Ontology, was developed to solve several problems with finding, browsing, and reasoning about GlyTouCan accessions, by computing semantic relationships, subsumption or degree of characterization, between the structures and compositions represented by glycan sequences. The representation of these relationships as an ontology, under the OBO Foundry banner, has facilitated use of GlyTouCan accessions (as GNOme terms) in other ontologies and standards efforts, and provided a way for curators to reference glycan compositions, topologies, and other partially defined structures described in manuscripts. The graphical user interface for exploring glycan structures and compositions by subsumption, the so-called GNOme Structure Browser, built on top of these relationships, provides an intuitive way to navigate the space of GlyTouCan accessions and their glycan structures to find those with a specific characterization of monosaccharide residues and glycosidic linkages. Finally, these relationships have enabled automated reasoning and propagation of annotations associated with more well-characterized structures to those that are less characterized and provided definitive semantic name to GlyTouCan accession mappings for easy lookup.

While GNOme does not cover all the accessions of GlyTouCan, such as those with less frequently used monosaccharides or those described with repeating substructures, we have found that GNOme is able to describe almost all non-repeating structures consistent with the SNFG monosaccharides and substituents, plus a substantial number of non-repeating structures that are not consistent with the SNFG.

GNOme is being used by other informatics efforts, including GlyGen, the Protein Ontology (PRO), and the Proteomics Standards Initiative (PSI), and is helping to bridge the gap between glycoinformatics efforts and the larger biomedical ontology and curation community, but more importantly, GNOme helps span the semantic gap between glycoinformatics data-resources and the glycobiology communities they serve.

## Supplementary Information

Below is the link to the electronic supplementary material.

Supplementary file1 (PDF 322 KB)

## Data Availability

All code and derived output files, ontologies, etc. are available from public GitHub repositories, and published on the internet at https://gnome.glyomics.org. The primary GNOme GitHub repository https://github.com/glygen-glycan-data/GNOme contains versioned releases, restrictions, the https://gnome.glyomics.org website, and the GNOme web-applications. The GitHub repository https://github.com/glygen-glycan-data/PyGly contains the Python code and shell scripts for retrieving GlyTouCan entries, parsing glycan sequence, computing glycan subsumption relationships, structuring the subsumption relationships as an OBO ontology, precomputing GNOme restrictions, and publishing GNOme releases.
