# Supplementary material for: GNOme, an ontology for glycan naming and subsumption
Source: Anal Bioanal Chem. 2025 Feb 8;417(10):1961–73. doi: 10.1007/s00216-025-05757-8 (PMC11961537; doi:10.1007/s00216-025-05757-8)
Supplement: Supplementary file 1 — Supplementary file1 (PDF 322 KB) [file 216_2025_5757_MOESM1_ESM.pdf]

GNOme, an Ontology for Glycan Naming and Subsumption

Wenjin Zhang[1], Michelle Vesser[1], and Nathan Edwards[1,2]

[1]Department of Biochemistry and Molecular & Cellular Biology,

Georgetown University Medical Center,

Washington D.C., USA

[2]Corresponding Author. Email: nje5@georgetown.edu, ORCID: 0000-0001-5168-3196

Supplementary Material

Contents

Glycan Subsumption Relationships 2

Structure Characterization Scores 4

SNFG Consistent Glycans 6

Glycan Subsumption Relationships

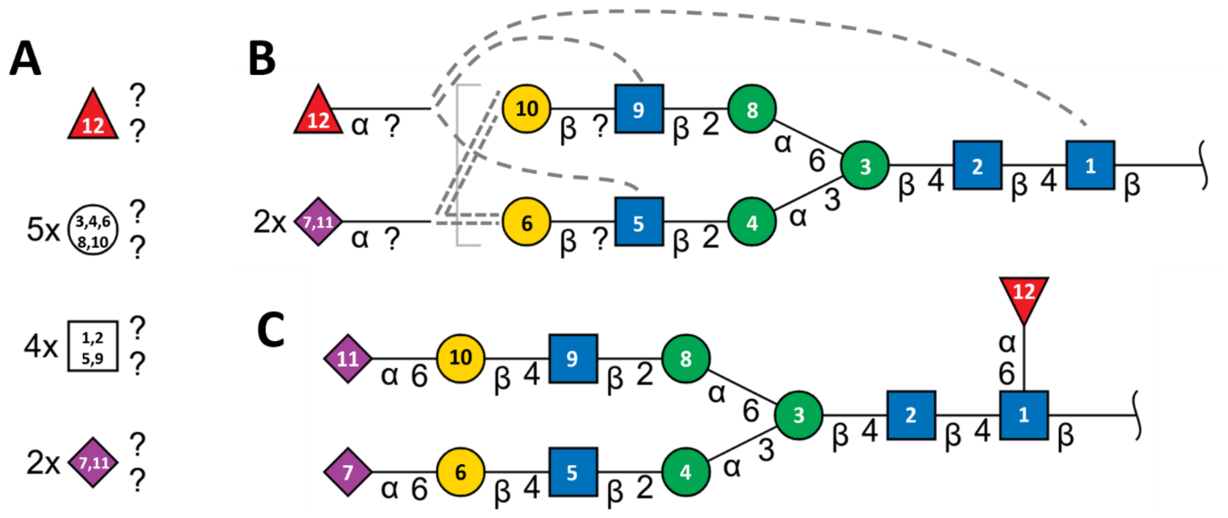

Figure S1: Three glycans, representing a composition (A), a structure with undetermined topology (B), and a fully defined structure (C). Subsumption aware perfect matching between monosaccharides is indicated by the numbers inside of each monosaccharide symbol. Monosaccharides shown with multiplicity represent multiple identical monosaccharides. Glycan B has its usual bracket augmented with dashed lines indicating the potential glycosidic links for these three undetermined monosaccharides. Double dashed lines are shown to represent the pair of potential glycosidic links for each of the two sialic acids.

In order to further elucidate the determination of subsumption relationships between glycans, we present some concrete examples of the major types of subsumption partial order determination. Figure S1 shows three glycans. Glycan A is a composition (GlyTouCan accession G45395BF). Glycan B is a glycan structure with undetermined topology (simplified version of GlyTouCan accession G22644UB with fewer possibilities for the undetermined glycosidic links). Glycan C is a fully defined structure (G56749GV). Glycan B has its usual bracket augmented with dashed lines indicating the potential glycosidic links for these three undetermined monosaccharides. Double dashed lines are shown to represent the pair of potential glycosidic links for each of the two sialic acids.

We denote the subsumption aware perfect matching between each of the monosaccharides in glycans A, B, and C by a number inside the monosaccharide symbol. Since

glycan A is a composition and glycans B and C are structures, this is sufficient to establish that A subsumes B and C. We note that due to the multiplicity and lack of stereochemistry information in the monosaccharides of A, there are many valid subsumption aware perfect matchings between the monosaccharides of A and B, and A and C; but only one such matching is required to establish subsumption. There are also multiple valid subsumption aware perfect monosaccharide matchings between B and C (though fewer, since Hex residues are resolved to Man and Gal), but some of these matchings do not result in valid corresponding link matchings.

When we determine the subsumption relationship between B and C, we must verify that the links between corresponding monosaccharides themselves satisfy the subsumption relationships. This can be seen in the β-4 link between monosaccharides 5 and 6 in C which is subsumed by the β-? link between monosaccharides 5 and 6 in B. Similarly for monosaccharides 9 and 10. The other links in the topologically determined part of B are matched with equality with the corresponding links in C, which leaves the potential links for the unplaced monosaccharides 7,11, and 12 to be checked. If we consider the α-6 Fuc link between monosaccharides 1 and 12 in C, it can be subsumed by the α-? Fuc potential link in B, leaving the potential α-? Fuc links to 12 from 5 and 9 unmatched. Similarly, the α-? NeuAc potential link in B to 7 from 6 subsumes the α-6 NeuAc link in C, and leaves the potential α-? NeuAc links to 7 from 11 unmatched. These unmatched potential links are OK, as long as all the links from C are subsumed by corresponding links in B. These final checks are sufficient to establish that B subsumes C. With the “shortcut” A subsumes C removed, since it can be inferred by transitivity, we can now see the subsumption hierarchy of these three glycans: $A \supset B \supset C$.

Structure Characterization Scores

A heuristic structure (lack of) characterization score between 0 and 10,000 is computed for each GNOme structure. Fully determined structures have characterization scores of 0. Base compositions have characterization scores of 10,000. Here we describe the approach to computing the structure characterization scores.

The score is determined from the normalized monosaccharide score and the normalized glycosidic linkage score, which are then combined using a weighted average and rescaled between 0 and 10,000. Define n to be the number of monosaccharides in the structure.

The single monosaccharide score penalizes missing anomeric center, ring information, configuration, and stem-type at two points each, and is rescaled between 0 and 10. The normalized monosaccharide score *mscore* first computes the sum of the single monosaccharide scores and then normalizes by the maximum possible value (10*n), rescaling between 0 and 1.

The single glycosidic linkage score *single_link_score(l)* penalizes multiple parent and child bond-types (unusual) two points each, and missing or multiple parent and child carbon bond positions at two and one points each, and is rescaled between 0 and 10. Next, for each non-reducing-end monosaccharide m, its parent-links are checked. If m has no parent links, its link-score is 10. If m has one parent link l (determined glycosidic linkage), its link-score is β**single_link_score(l)*. If m has more than one parent link (undetermined glycosidic linkage) the average *single_link_score* of the parent links *average_single_link_score* is determined. The proportion of monosaccharides represented as parent links is then added as an undetermined monosaccharide *undet_mono_prop*. The final link-score for m is then:

α**average_single_link_score* + (1- $\underline{\alpha}$)*10**undet_mono_prop*.

The normalized glycosidic linkage score *lscore* sums the link-score for each non-reducing-end monosaccharide *m* and normalizes by the maximum possible value (10*(*n*-1)), rescaling between 0 and 1. For structures with no glycosidic links (compositions), the normalized glycosidic linkage score is 1.

Once the normalized monosaccharide score *mscore* and the normalized glycosidic linkage score *lscore* are computed, the structure characterization score can be determined as β**mscore* + (1- β)**lscore*. The parameters α and β are set to 0.65 and $\alpha/(1+\alpha)$ respectively.

SNFG Consistent Glycans

SNFG Consistent Glycans are then those that consist entirely of monosaccharides and substituents consistent with the Symbolic Nomenclature for Glycans (SNFG) documentation provided on the site Symbol Nomenclature for Glycans (SNFG) site. The Symbol Nomenclature section Table 1 provides a list of monosaccharides, in their most common configuration, and by virtue of its links to PubChem, the WURCS sequence and GlyTouCan accession for a single monosaccharide glycan structure consisting of the monosaccharide can be determined. The GNOme website provides a table of monosaccharides plus this derived information at https://gnome.glyomics.org/SNFG. Table 3 of the SNFG Symbol Nomenclature section provides a list of monosaccharide substituents and abbreviations.

The WURCSFilter also provides a SNFG monosaccharide and substituents list, with WURCS monosaccharide residues codes and WURCS substituent MAP strings. We have correlated the WURCS monosaccharide residue codes between these resources and the SNFG site and to ensure they correspond.

SNFG consistent glycans, then, are those with WURCS sequences that consist entirely of SNFG monosaccharides, optionally with additional SNFG substituents attached to the monosaccharides, and optionally with unattached SFNG substituents.

Define S_{SNFG} to be the set of SNFG substituent WURCS MAP strings and S^*_{SNFG} to be the set of SNFG substituent WURCS MAP strings plus the substituent WURCS MAP strings from M_{SNFG} SNFG monosaccharides. We note that there *are* substituents in the SNFG monosaccharides that are not in the SNFG substituent list, so S^*_{SNFG} is a superset of S_{SNFG}.

For any WURCS sequence then, we consider each monosaccharide and unattached substituent (represented in the links section of the sequence) in turn.

For any unattached substituent WURCS MAP string, we check for membership in S_{SNFG}. If an unattached substituent is not in S_{SNFG}, it is not SNFG consistent and neither is its glycan.

For monosaccharide WURCS residue codes *m*, we first check its substituents' WURCS MAP strings for membership in S^*_{SNFG}. If any of its substituents is not in S^*_{SNFG}, then the monosaccharide is not SNFG consistent and neither is its glycan.

Next, we consider each of the SNFG monosaccharides m_{SNFG} in decreasing order based on the number of substituents. We consider all possible matchings of *m*'s substituent WURCS MAP strings with m_{SNFG}'s substituent WURCS MAP strings that match all of m_{SNFG}'s substituents. If no such matching can be found, we consider the next SNFG monosaccharide. For any such matching, the unmatched substituent WURCS MAP strings from *m* are checked for membership in S_{SNFG}. If any unmatched substituent is not in S_{SNFG}, then *m* is not SNFG consistent and neither is its glycan. Next, *m* with unmatched substituents removed m' is checked to determine if it subsumes m_{SNFG}. Since the SNFG monosaccharides do not indicate anomeric configurations, we remove any anomeric configuration and alditol modifications from m' before checking for subsumption. If m' subsumes m_{SNFG} (and its removed substituents are in S_{SNFG}) then it is considered SNFG consistent. If not, we consider the next SNFG monosaccharide.

If after considering all SNFG monosaccharides, the monosaccharide is not found to be SNFG consistent, then is its glycan is not SNFG consistent.

The SNFG consistency of each monosaccharide and unattached substituent in each GlyTouCan WURCS sequence is provided with each GNOme release https://gnome.glyomics.org/data/glytoucan_snfg.txt.
